# Host cell factors involved in classical swine fever virus entry

**DOI:** 10.1186/s13567-023-01238-x

**Published:** 2023-12-01

**Authors:** Yaneysis Lamothe-Reyes, Maximiliano Figueroa, Oliberto Sánchez

**Affiliations:** 1 Laboratory of Molecular Biophysics, Department of Biochemistry and Molecular Biology, Faculty of Biological Sciences, University of Concepcion, Concepcion, Chile; 2 Laboratory of Recombinant Biopharmaceuticals, Department of Pharmacology, Faculty of Biological Sciences, University of Concepcion, Concepcion, Chile

**Keywords:** CSFV, receptor, CD46, HS, Laminin receptor, ADAM17, entry

## Abstract

Classical swine fever virus (CSFV) is an ancient pathogen that continues to pose a threat to animal agriculture worldwide. The virus belongs to the genus *Pestivirus* and the family *Flaviviridae*. It causes a multisystemic disease that affects only pigs and is responsible for significant economic losses. CSFV infection is probably a multistep process that involves the proteins in the virus envelope and more than one receptor in the membrane of permissive cells. To date, the cellular receptors essential for CSFV entry and their detailed functions during this process remains unknown. All the viral envelope proteins Erns, E1 and E2 are involved in the entry process to some extent and the experimental approaches conducted until now have helped to unveil their contributions. This review aims to provide an overview of current knowledge on cellular molecules described to be involved in CSFV entry, including complement regulatory protein 46 (CD46), heparan sulphate (HS), Laminin receptor, Integrin ß3, Annexin II, MERKT and ADAM17. This knowledge would not only help to understand the molecular mechanisms involved in pestivirus infection, but also provide a rational basis for the development of nonvaccinal alternatives for CSFV control.

## Introduction

Classical swine fever virus (CSFV) is an ancient pathogen responsible for classical swine fever (CSF), one of the most lethal diseases affecting domestic pigs and wild boar [1, 2]. CSF causes massive economic losses and is a threat to animal health, which is the main reason for its mandatory notification to the World Organization for Animal Health (WOAH) [3]. CSFV is a single-stranded, positive-sense enveloped virus belonging to the genus *Pestivirus* within the family *Flaviviridae*, which also includes the genera *Flavivirus*, *Hepacivirus*, and *Pegivirus* [4, 5]. Other well-known pathogens included in this genus *Pestivirus* are the bovine viral diarrhea virus-1 (BVDV-1), BVDV-2, and border disease virus (BDV) [2]. CSF virions have icosahedral symmetry and are 40–60 nm in diameter. Its genome of approximately 12.3 kb contains untranslated regions (UTR) at the 5′ and 3′ ends, and a large open reading frame (ORF) encoding a single polyprotein. After co- and post-translational processing by viral and host proteases, the polyprotein matures into 4 structural and 8 non-structural proteins [6–8]. The non-structural proteins (Npro, p7, NS2, NS3, NS4A, NS4B, NS5A and NS5B), except for Npro, are distributed toward the 3’ end of the genome. They are encoded by an amino acid sequence that is highly conserved among members of the genus *Pestivirus* and assist mainly in viral replication and immune evasion [9]. Structural proteins include C, Erns, E1 and E2, which are mainly involved in virus assembly. Erns, E1 and E2 are located on the surface of the virion while C is associated with the capsid [7].

CSFV enters the host through the mucous membranes of the oronasal cavities and initially infects the epithelial cells of the tonsillar crypts, spreading throughout the body via the blood and lymphatic circulation and subsequently invading the lymphoid tissue [10]. In a next step, the virus is transported to the regional lymph nodes and enters the efferent blood capillaries, resulting in viremia [11]. The virus then replicates in the bone marrow and secondary lymphoid organs, including the spleen, lymph nodes, and small intestinal lymphoid structures [12]. Parenchymatous organs are invaded late in the viremic phase. CSFV has a distinctive tropism for cells of the immune system, causing severe leukopenia associated with apoptosis of leukocytes in the thymus, spleen, lymph nodes, and bone marrow of infected pigs [13–16]. The ultimate outcome of viral infection is generally associated with a complex and diverse host response to the virus.

Interactions between the envelope proteins of a virus and cellular receptors are crucial in the infection process [17]. These interactions can facilitate the attachment of the virus to the cell without the subsequent membrane fusion and entry, or they can induce the conformational changes necessary for these events to occur. The receptors involved in the first of these interactions are known as attachment receptors and play an important role in the infection of certain cells by increasing entry efficiency. However, it is the presence of entry receptors that determines whether a particular cell type is susceptible to infection [18]. The cellular mechanisms leading to CSFV entry requires a deeper study. The virus-host interactions that take place during infection are relevant for the discernment of the mechanisms involved in this process and to open new opportunities to develop novel therapeutic approaches. The aim of this review is to provide an overview of the current knowledge related to the interactions between CSFV and the host cell factors involved in its entrance.

## Viral proteins involved in CSFV entry

The infection process of CSFV, like that of other members of the *Flaviviridae* family, is probably a complex multistep process not fully understood. Cell entry of *pestiviruses* in general, and of CSFV in particular, is probably one of the least comprehended events. It has been established that CSFV enters PK15 cells through a receptor-mediated endocytosis mechanism dependent on clathrin, cholesterol, dynamin, and the Rab5 and Rab7 proteins [19]. A similar mechanism was demonstrated for BVDV internalization, including the need of low-pH conditions [20, 21]. Likewise, it seems that CSFV can also employ different endocytic pathways to infect different cells, as it showed to relay in caveolin to infect porcine alveolar macrophages [22]. CSFV surface proteins are involved in the initial interaction with the membrane receptors. Erns facilitates initial cell attachment [29], while interaction of E2 with one or more receptors mediates entry. After entry, fusion of the viral and cellular membranes is mediated by the E1 and E2 glycoproteins in a pH-dependent process [28], in which the E2 peptide _129_CPIGWTGVIEC_139_ is thought to be involved [30]. Once the genetic material is released into the cytosol, the pathogen hijacks the cellular machinery to replicate its genome. The study about the contribution of the CSFV envelope glycoproteins is relevant to understand the mechanisms that the virus employs during infection.

### Erns

Erns is a unique viral protein that can be anchored to the membrane or secreted into the extracellular environment [23]. Both forms of the protein are precisely balanced. The anchored protein contains a long amphipathic helix at its C-terminus that is slightly tilted into the membrane. This differs from the transmembrane domain of E2 and E1 [24, 25]. The soluble form possesses RNase activity, which may be involved in the regulation of RNA synthesis in infected cells, the induction of immunosuppression in the host, and the attenuation of virulence in a virulent background [23, 26]. In the virion, the protein has a molecular weight of 42–48 kDa, but is usually found as a homodimer of about 100 kDa, established by the carboxyl-terminal cysteine C171 [27]. The protein is highly glycosylated with approximately 50% of its mass consisting of N-linked glycosyl groups [28].

Erns is essential for pestivirus replication but dispensable for infection, as shown by experiments with pseudotyped retroviruses carrying pestivirus glycoproteins [29]. It is thought that the protein may mediate initial attachment to cells by interacting with a widely expressed surface molecule [29]. This was suggested by the fact that Erns of a CSFV vaccine strain also binds to the surface of cells from different species [30]. Hulst et al. [31] investigated the role of GAGs in the initial binding of CSFV to cells. They concluded that the interaction of CSFV Erns with membrane-associated HS facilitates the binding of the virus to the cell surface. This finding is confirmed by the inhibition of binding after propagation in porcine PEDSV.15 cells [32] or in the presence of the drug DSTP 27 [33]. It appears that the increased binding to glycosaminoglycans is a result of cell culture adaptation. The change S476R in the C terminus of Erns proved to be sufficient to transform a HS- independent virus into a virus that uses HS as an Erns receptor [31]. It is thought that electrostatic forces are the cause of this interaction, although HSPG might interact more specifically with the BVDV Erns as it was proposed in another study [34]. Several other data suggest that Erns might interact with a more specific receptor, maybe the Laminin receptor [35]. Its ability to induce neutralizing molecular antibodies capable of blocking infection of HS-independent genotypes or to exert cytotoxic action on lymphocytes in its unbound state favored this idea [36–38]. However, until now its exact contribution to virus infection and entry remains to be discovered.

### E1

E1 is the least characterized of the CSFV structural proteins, and its structure and function require further investigation [23]. It appears to be buried in the membrane, as no antibodies to the protein have been detected in infected animals [39]. E1 is a 33 kDa protein with a transmembrane anchor that forms disulfide-linked heterodimers with E2 on the virus particle [40]. E1 and E2 are the only envelope glycoproteins required to mediate fusion of pseudotyped viruses [29]. The protein has been implicated in the fusion process during viral infection. A stretch of hydrophobic amino acids (57–85) in E1 has been proposed as the peptide responsible for fusion of viral and cellular membranes [41]. However, E1 is only about half the size of E2, and E2 is an elongated protein; thus, E1 would have to be very long and thin to bridge the distance to the target membrane after E2 has established receptor contact [23]. The study of the protein in hepatitis C virus, another member of the *Flaviviridae* family, suggests that E1 might play a more important role than previously thought in the flavivirus life cycle. Mutations in E1 affect the infectivity of pseudoparticles carrying the HCV glycoproteins and modulate the binding of these particles to CLDN1-expressing cells [42]. These findings support the idea that mutations in E1 might abolish a critical interaction between E1 and CLDN-1 or affect how E2 interacts with cell entry factors. In this regard, it has been observed that mutations in E1 can affect E2-CD81 interaction, indicating that E1 plays a role in modulating the receptor binding capacity of E2 [42]. In another study it was found that mutations T213A and I262A in the protein generated attenuation and shifted the virus receptor dependence from Claudin-1 to Claudin-6 in the target cell line [43].

Studies have also demonstrated the importance of E1 for virulence. An insertion of 19 amino acids into the C-terminus of E1 attenuates the highly virulent strain Brescia [43]. In silico analysis predicted that the insertion introduces an alpha helix and turns near the E1/E2 cleavage site. This change, as described by the authors, may directly affect a specific virulence determinant on E1 or alternatively alter its ability to interact with E2 or other viral proteins critical for viral uptake, cell tropism, or viral spread in the host. It has also been reported that the N6A, N19A, and N100A substitutions cause infertility and attenuation in the BICv strain [44].

### E2

E2 is the most immunogenic protein of the virus and induces a neutralizing antibody response capable of conferring protection [45, 46]. As the main target for neutralizing antibodies, E2 alone can induce protection in animals. This and the fact that E2 is responsible for species tropism has been exploited in vaccine production. The glycoprotein is essential for the life cycle of the pathogen and an important virulence determinant, as virus mutants with partial or complete deletions of E2 are not viable [47, 48]. It is also responsible for the interaction with the cellular receptor that mediates entry determining viral tropism. E2 is anchored to the viral membrane by a hydrophobic sequence in its C-terminus [49]. It can be found as a homodimer of about 100 kDa, but mostly forms heterodimers with E1 of about 75 kDa, stabilized by a disulfide bond [2]. Within the viral genome, the N-terminal half of E2 is one of the most variable regions, so phylogenetic analyses based on 190 nucleotides of this region are used for virus classification [50]. The use of monoclonal antibodies (mAbs) directed against the E2 protein of the Brescia strain of CSFV allowed the identification of 4 antigenic domains: A, B, C and D, at the N-terminal end of the protein [51]. In a model built from the three-dimensional structure of the E2 of this strain, 4 antigenic domains (A, B, C and D), the potential glycosylation sites and the present disulfide bridges are identified. Domain A has 3 subdomains: A1, A2 and A3, of which the first 2 are highly conserved among different virus strains, while A3 is more variable. Only the A1 subdomain together with the B and C domains induce neutralizing antibodies, but the sequences corresponding to the B and C domains are less conserved [52]. The antigenic sequence _829_TAVSPTTLR_837_, located in the A/D domain of the protein, has been defined as highly conserved among the different CSFV variants, but not among the different pestiviruses. This sequence is also the target of neutralizing antibodies [53]. A study using a 30-amino acid peptide library whose sequences together spanned the entire protein identified the sequence _830_AVSPTTLRTEVVKTFRRDKPFPHRMDCVTT_858_ as being involved in virus-cell binding [54].

However, even when research on CSFV glycoproteins has been focused mainly on E2 because all the above-mentioned reasons, it is worth of consideration the cooperating role of E1 during the entry process. It might be, as has been observed in the case of HCV, more important than we have previously thought.

## Cellular proteins involved in CSFV entry

Identifying the cellular proteins that mediate virus attachment and entry is critical to understanding virus entry. Unfortunately, the pestivirus entry process remains poorly understood, although previous studies have shown that CSFV infects PK15 cells by binding to several membrane proteins followed by clathrin-dependent endocytosis [19]. Caveolin-dependent entry has also been reported in porcine alveolar macrophages [22]. To date, seven cellular receptor molecules have been described for CSFV, namely heparan sulfate (HS), complement regulatory protein 46 (CD46), low-density lipoprotein (LDL) receptor, Integrin ß3, Annexin 2, MERKT, Laminin receptor and a disintegrin and metalloproteinase 17 (ADAM17). The main results related to the identification of these molecules and their relevance in the infection are described in detail in the next paragraphs and summarized in Table 1.

**Table 1:** **Cellular factors/proteins involved in CSFV entry**

Cellular factor/protein	Functions	CSFV partner	References
CD46	- Acts as a complement regulator - BVDV receptor	*Probably* E2	[33, 60]
ADAM17	- Intervenes in the processing of transmembrane proteins	E2	[65]
Heparan sulfate	- Prevents degradation of proteins/substances - Acts as an endocytosis receptor - Is involved in the attachment of multiple virus	Erns	[31, 33, 68]
Laminin receptor	- Cellular receptor - Is involved in adeno-associated virus infection	Erns	[35]
Integrin ß3	- Is involved in proliferation and migration of endothelial cells - Acts as receptor/co-receptor for other viruses	?	[77, 79]
Annexin II	- Participates in membrane trafficking - Has ion channel activity - Participates in DNA replication - Acts as a virus receptor	E2	[81, 83, 84, 86–88]
MERKT	- Is involved in the phagocytic clearence of apoptotic cells - Potentiates virus infection	E2	[89–92]

### Complement regulatory protein (CD46)-46

Membrane cofactor protein, also known as CD46, is a type I transmembrane protein that serves as a complement regulator [55]. It is expressed in human cells as 4 isoforms derived from alternative splicing of a single gene of approximately 46 kb [56]. CD46 protects host cells from complement attack by acting as a factor I cofactor in the proteolytic inactivation of C3b and C4b [57]. In pigs, the protein is present in cells of epithelial and endothelial origin, fibroblasts and circulating cells and, in contrast to its human counterpart, is abundantly expressed in erythrocytes [55, 58]. Bovine CD46 was identified as the receptor for BVDV after monoclonal antibodies directed against the receptor inhibit viral infection [59, 60]. Furthermore, expression of bovine CD46 in porcine cells increased their susceptibility to BVDV infection [60]. Further studies revealed that peptides _66_EQIV_69_ and _82_GQVLAL_87_, located on the antiparallel ß-sheet of CD46 CCP 1, were critical for receptor interaction with BVDV [61]. A study by Hulst et al. [30] suggested that BVDV and CSFV use a homologous receptor because the soluble E2 protein of CSFV could inhibit infection by both viruses. Considering that pestiviruses are structurally and antigenically closely related, the involvement of porcine CD46 in CSFV infection was investigated.

Dräger et al. [33] incubated virus-permissive cells with a mixture of monoclonal antibodies directed against porcine CD46. The cells were then infected with Roesrath, a moderately virulent variant of CSFV and analyzed by immunofluorescence for evidence of viral infection. The results suggested that CD46 is involved in CSFV infection, since blockade of the protein results in an almost complete inhibition of viral infection. However, when the experiment was repeated using the Roesrath variant, which was subjected to 30 passages in culture, blockade of CD46 had less effect on infection. The authors demonstrated that this was due to the preferential use of HS after adaptation to cell culture, which has been previously observed for CSFV and other viruses [62]. Previous studies by Hulst et al. [31] had shown that in the case of CSFV, after adaptation to culture in SK6 cells, a change from serine to arginine at position 476 of the Erns protein favors binding to HS. It is not known whether this mutation in Erns, which is also sufficient for binding, arises to compensate for any mutation(s) in E2 that reduce(s) the affinity for its own receptor. Since then, it has been assumed that CD46 is one of the receptors involved in CSFV infection, that it interacts with E2. Conversely, a recent study questioned the involvement of CD46 in CSFV infection [63]. Using virus isolates of different virulence and representing genotypes 1 and 2 of the virus, CD46 was not found to be involved in infection. Knockout of the protein did not alter viral infection. Even when some experiments suggest that porcine CD46 plays an important role in CSFV infection, the absence of complete inhibition of the infection after using antibodies directed against the protein, suggests the involvement of other entry factors.

### A disintegrin and metalloproteinase 17 (ADAM17)

The tumor necrosis factor-α-converting enzyme (TACE) usually known as ADAM17 is a single-pass transmembrane metalloproteinase responsible for the processing of many transmembrane proteins. Preliminary studies indicate that it might also be involved in BVDV infection [64]. CRIB cells, which are resistant to infection with BVDV do not express functional full-length ADAM17 mRNA and have two defective alleles of the protein. When ADAM17 was provided in trans in CRIB-1 cells, their resistance to infection with a diverse array of pestivirus (BVDV-1, HoBiPeV, CSFV, LindaV) was nearly completely reverted. Nevertheless, the susceptibility to infection or the propagation efficiency never reach the levels observed in MDBK cells, a cell line used in BVDV infection experiments [64]. This indicates that the effect of additional factors involved in the infection of CRIB cell resistance must be investigated.

Yuan et al. [65] have shown that the protein is also related to CSFV infection of permissive cells. The knockout of the protein in PK15 cells blocked the binding of soluble E2 protein and the entry of CSFV pseudotyped particles (CSFVpp) as well as cell culture grown CSFV. The interaction of the E2 protein and ADAM17 takes place through the metalloproteinase domain. A protein mutant lacking ADAM17 intracellular domain completely reestablished the infection in ADAM17-KO cells, suggesting that the protein serves as an attachment factor during CSFV entry and not as a virus internalization receptor. The sequence aa301-345 in the metalloproteinase domain involved in virus-host recognition is identical in pig, human and mouse. This is a possible explanation of why mouse and human ADAM17 could confer permissiveness of CSFVpp to ADAM17-KO cells as efficiently as pig ADAM17. This indicates that ADAM17 is not a host determinant of CSFV infection, and the infection process might require the involvement of other proteins.

### Heparan sulfate

Heparan sulfate proteoglycans (HSPG) are cell surface proteins covalently linked to glycosaminoglycan (GAG) chains of heparan sulfates (HS), an unbranched sulfated anionic polysaccharide [66]. HSPGs are present at the cell surface membrane and their multiple functions include preventing the degradation of cytokines, chemokines, growth factors, and morphogens, and serving as endocytosis receptors for extracellular molecules and other cellular receptors. The highly sulfated GAG chains of HPSG provide the global negative charge sufficient to electrostatically attract the basic residues of viral surface glycoproteins or the viral capsid proteins of non-enveloped viruses. These interactions, even the weak ones, can increase the virus concentration at the cell surface and facilitate the likelihood of binding a more specific entry receptor [67].

Many viruses, ranging from natural isolated to laboratory strains, have shown a HSPG dependance during infection [66]. For others, the use of HSPG is a result of cell culture or intra-host adaptation. This seems to be the case of CSFV. According to the investigations carried out by Hulst et al. [31] and supported by other studies [33, 68], CSFV is able to use HS as an attachment factor after adaptation to cell culture. Treatment of SK6 cells with heparinase I and heparin affected the infection of those virus clones that had been amplified in cell culture, but not of newly isolated clones. As it has been previously described, a mutation of Serine to Arginine in Erns at the position 476 of the CSFV genome, is responsible for this dependency. The substitution of Ser for Arg increases the net positive charge of this region, facilitating the electrostatic interaction of proximal or more distant amino acids of the protein with the negatively charged HS [31]. Although, the possibility of more specific interaction with HS is a plausible idea, as it has been reported to take place during the infection of Herpes simplex virus type 1 (HSV-1) [69]. In this case, the 3-*O*-sulfation of specific glucosamine residues in heparan sulfate generates binding sites for the viral gD protein or the expression of other previously identified gD-binding receptors.

The role of HS for CSFV infection in vivo is a reasonable concern considering the influence of cell culture adaptation in this interaction. Hulst et al. [68] generated HS independent (Ser476) or dependent (Arg476) mutants from a virulent strain of CSFV to conduct infection experiments with pigs. Further reisolation of these viruses showed that the HS-independent recombinant ones were able to infect cultured and primary swine kidney cells by an HS-dependent mechanism. No mutations were observed in the Erns, E1, and E2 genes of this virus, suggesting that the surface properties of CSFV generated in pigs, carrying Ser476 in the C terminus of Erns, were distinct from those of genetically identical virus produced in cell culture.

More studies are needed to elucidate the role of the molecule in the virus infection in vitro and in vivo. HS can function as an independent attachment factor or work coordinately with another more specific cell receptor to mediate CSFV entry considering that, in most cases, binding of viruses to HS is not sufficient to enter the host cell.

### Laminin receptor

Laminin receptor (LamR) is a protein involved in the specific interactions with Laminin. It is expressed in the cell in two forms: a cytoplasmic protein of 37 kDa and a membrane protein of approximately 67 kDa [70, 71]. In mammalian cells, LamR acts as a cellular receptor for the cellular prion protein (PrP) [72], and it mediates the infection of adeno-associated virus serotypes 8, 2, 3, and 9 [73]. It has also been identified as a cellular receptor for Dengue virus, another member of the *Flaviridae* family [74, 75]. Chen et al. [35] found that the protein is involved in the attachment of CSFV to the cell after screening pooled siRNAs against porcine membrane proteins. Anti-LamR antibodies, soluble laminin or LamR before virus incubation decreased CSFV infection. The membrane protein seems to interact with Erns after a co-immunoprecipitation assay. The receptor expression did not confer susceptibility to infection in nonpermissive cells, which might indicate that it acts as an attachment factor. This hypothesis was validated by the fact that LamR expression was sufficient to increase the binding of CSFV virions to both permissive and nonpermissive cells. The researcher suggested that LamR functioned as an alternative pathway to the HS pathway. If there is abundant HS on the cell surface, increasing the expression of LamR does not enhance the infection rate. Therefore, LamR is involved in CSFV attachment but it not the receptor responsible for its.

### Integrin ß3

Integrin ß3 is a member of the integrins superfamily present at the membrane of a wide range of cells. It is a heterodimer composed of noncovalently bound a and b subunits and has been classified into this subfamily by its ß subunit. Structurally, each subunit is a transmembrane glycoprotein whose N-terminal domains combine to form the ligand-binding site. Integrin ß3 is known to be important in endothelial cell migration, vascular biology, and tumor angiogenesis. It is also an important cell receptor mediating complex outside-in signaling, probably the reason why many viruses use it as a receptor, co-receptor, or a key molecule for infection [18, 76, 77]. Many of these virus-integrin interactions are dependent on the arginine-glycine-aspartic acid (RGD) cell-adhesion motif The RGD motif can interact with over half of the more than 20 known integrins [78].

Li et al. [79] investigated the role of integrin β3 during CSFV infection and proliferation. The authors found a positive correlation between CSFV proliferation in swine testicles epithelial cells (ST) and the high amount of integrin β3 in these cells. In addition, the amount of CSFV proliferation decreased in integrin β3-funtionally blocked cells as well as in integrin β3-deficient cells. However, even when this study suggests that integrin β3 could be a promising receptor for CSFV, additional studies are needed to evaluate if the protein is indeed related with virus attachment or entry.

### Annexin II

Annexin II is a member of the annexin gene family with binding sites for Ca^2+^, phospholipids, and F-actin in its core domain. It has also been reported that its COOH-terminal region binds heparin and plasminogen [80–82]. The protein exists as a monomer (p36) or a heterotetramer formed from two p36 and two p11(S100A10) subunits. It has been implicated in exocytosis and endocytosis pathways, as well as in ion channel activity and stimulation of DNA replication [81]. Annexin II traffic to the cell surface is conducted by an unknown mechanism [80]. It has been identified as a receptor for CMV [83] and respiratory syncytial virus [84], and a co-factor for macrophage human immunodeficiency virus (HIV)-1 infection [85]. Some studies have assessed the participation of Annexin II in CSFV infection. The genomic expression of the protein was upregulated in CSFV-infected PK-15 cells [86] and the treatment of PK-15 cells with Anx2-specific polyclonal antibody prior to virus infection significantly inhibited CSFV multiplication [87]. In pig peripheral blood leukocytes Annexin II expression was also upregulated after CSFV in vivo infection [88]. In this study the protein co-localized in the cytoplasm of infected PK-15 cells with CSFV glycoprotein E2 after confocal laser-scanning microscopy. This finding suggests that annexin II may interact with certain viral proteins during CSFV replication and thereby promote the virus cycle.

### MERTK

MERTK is a protein tyrosine kinase included in the TAM (TYRO3, AXL, and MERTK) family. The tyrosine kinases are related with the phagocytic clearance of apoptotic cells and the antagonism of innate immune responses. The interaction of TAM receptors with different viruses has been related with viral infection potentiation. The entry mechanisms facilitated by this family are diverse depending on the member and probably also the virus. For example, AXL enables Zaire Ebolavirus (ZEBOV) entry by macropinocytosis [89] while mediating the entry of dengue virus (DENV) into host cells via the clathrin-dependent endocytosis pathway [90, 91]. An analysis of transcriptomic data describing gene expression profiles on PK-15 cells after CSFV infection revealed that MERTK could be involved in this process [92]. CSFV protein expression was significantly reduced by MERKT-RNA interference (RNAi) screening. Furthermore, either anti-MERTK antibodies or soluble MERTK ectodomain could reduce CSFV infection in PK-15 cells in a dose-dependent manner. It seems like the protein interacts with CSFV glycoprotein E2 during the entry process. This was indicated by its coimmunoprecipitation with E2 but not Erns and surface plasmon resonance (SPR) analysis of the ectodomains (ED) of E2 and MERTK. The equilibrium dissociation constant (KD) value between MERTK^ED^-His and E2^ED^-His was 1.629 µM. Correspondingly, the colocalization of MERTK and E2 was confirmed in HEK293 T cells, which transiently overexpress the two proteins. Post virus entry analysis showed that MERTK downregulates of mRNA expression of IFN-β and promotes CSFV infection. Interestingly, the soluble MERTK ectodomain could also reduce the infection of bovine viral diarrhea virus (BVDV), another pestivirus.

## Current issues and future challenges

CSFV is an interesting pathogen from a molecular, sanitary, and economic point of view. Investigations conducted over the last two decades have helped to elucidate some of the intriguing features of this virus and have contributed significantly to our view of the virus-host interplay. Although much is known about the events that occur after entry, our understanding of the mechanisms involved in viral entry needs to be further explored. Many questions remain: What is the key factor involved in CSFV entry? Is the host cell receptor(s) used by CSFV the same for all cell lines? Are the in vitro results a true reflection of what happens in vivo?

In the study of CSFV, some considerations may help to understand and focus future investigations. First, this virus, like hepatitis C virus (HCV), another member of the family, may use a different type of receptors and entry mechanisms [93–96]. This strategy is thought to facilitate viral infection in the absence of a receptor [35]. Second, even though Erns and E2 have been the more studied proteins to date, a closer look at E1 may reveal previously undescribed contributions. Finally, the combination of new technologies such as high-throughput gene sequencing, gene editing, and genome-based functional screening would be extremely helpful in the investigation of new molecules.

Some studies have already discovered many genes that are up- or down-regulated after CSFV infection, including the SR-BII gene, TYRO3, CD97, and CD69. For example, SR-BII has been found to mediate the internalization of hepatitis C virus (HCV) into cells through its interaction with HCV soluble E2 envelope glycoprotein. Its upregulation after CSFV infection suggests that it may also be involved in this process [88]. The study of these genes and the possible relationship between the receptors identified so far and their possible membrane partners is also part of the strategy to elucidate the CSFV entry mechanism.

## Conclusion

In summary, the experimental evidence available so far indicates that CSFV entry into the cell, at least in vitro, is mediated by Erns and E2 proteins. Interaction of Erns with HS or the Laminin receptor could facilitate the initial binding of the virus to the cell. Subsequently, interaction of E2 with various molecules on the membrane may lead to specific binding to the receptor that mediates entry. The proteins involved could be different depending on the virus genotype and cell type. Nevertheless, the key receptor involved in virus entry remains to be further investigated and will help us better understand the viral tropism.
